# Patellar Tendon Reconstruction Using Tibialis Posterior Allograft for Treatment of Patellar Tendon Rupture After Bone-Patellar Tendon-Bone Anterior Cruciate Ligament Reconstruction

**DOI:** 10.31486/toj.23.0104

**Published:** 2024

**Authors:** Andrew Renshaw, W. Evans Few, Bhumit Desai, Brian Godshaw, Deryk Jones

**Affiliations:** ^1^ Department of Orthopedic Surgery, Ochsner Clinic Foundation, New Orleans, LA; ^2^ The University of Queensland Medical School, Ochsner Clinical School, New Orleans, LA

**Keywords:** *Anterior cruciate ligament*, *bone-patellar tendon-bone grafts*, *patellar ligament*

## Abstract

**Background:** Bone-patellar tendon-bone (BPTB) autografts are often used to treat anterior cruciate ligament (ACL) tears in young, highly active patients. These grafts are robust and provide adequate stability, allowing for return to sport and optimal functional outcomes in athletes. Patellar tendon rupture following BPTB ACL reconstruction is rare and can be difficult to treat.

**Case Report:** A 19-year-old collegiate wrestler injured his left knee during a match. On evaluation 7 days after the injury, he was found to have increased anterior translation of the tibia on Lachman testing and an abnormal pivot shift. Magnetic resonance imaging demonstrated a complete tear of the ACL, and he successfully underwent a BPTB ACL reconstruction without complication. He progressed appropriately in the acute postoperative period. Six weeks after his index surgery, the patient reinjured his left knee and was diagnosed with a patellar tendon rupture. The previously reconstructed ACL was intact. A posterior tibialis tendon graft was used to repair the patellar tendon via a transosseous tunnel in the tibial tuberosity. The patient's recovery was complicated by a superficial wound that resolved with treatment. He achieved full range of motion and was able to return to sport.

**Conclusion:** No technique for treating patellar tendon rupture following BPTB ACL reconstruction has been widely accepted. The treatment of this injury is left to the preference of the surgeon. This case demonstrates that tibialis posterior allografts are a viable option for the treatment of such injuries.

## INTRODUCTION

Although anterior cruciate ligament (ACL) injury is one of the most common sporting injuries,^1^ conjecture remains regarding the most appropriate graft choice for ACL reconstruction. The commonly used bone-patellar tendon-bone (BPTB) graft is thought to provide better stability than a hamstring autograft but with higher morbidity.^2^

Patellar tendon rupture following BPTB ACL reconstruction is an uncommon complication, with studies demonstrating occurrence in 0.06% to 0.24% of cases.^3,4^ Patellar tendon rupture commonly occurs in the early postoperative period from a slip and fall accident,^3-8^ and case reports have described various locations of tendon rupture.^3-16^ The majority of these injuries have been complete distal or proximal ruptures, avulsions, or Z-type ruptures with the medial side of the tendon tearing proximally and the lateral side tearing distally. Only 2 papers^7,14^ described midsubstance tears, as detailed in this report. Different surgical techniques for reconstruction have been reported, depending on the location of the tear. This report illustrates the successful use of a tibialis posterior allograft for the reconstruction of a midsubstance patellar tendon rupture following patellar tendon harvesting for ACL reconstruction.

## CASE REPORT

**Figure 1.**
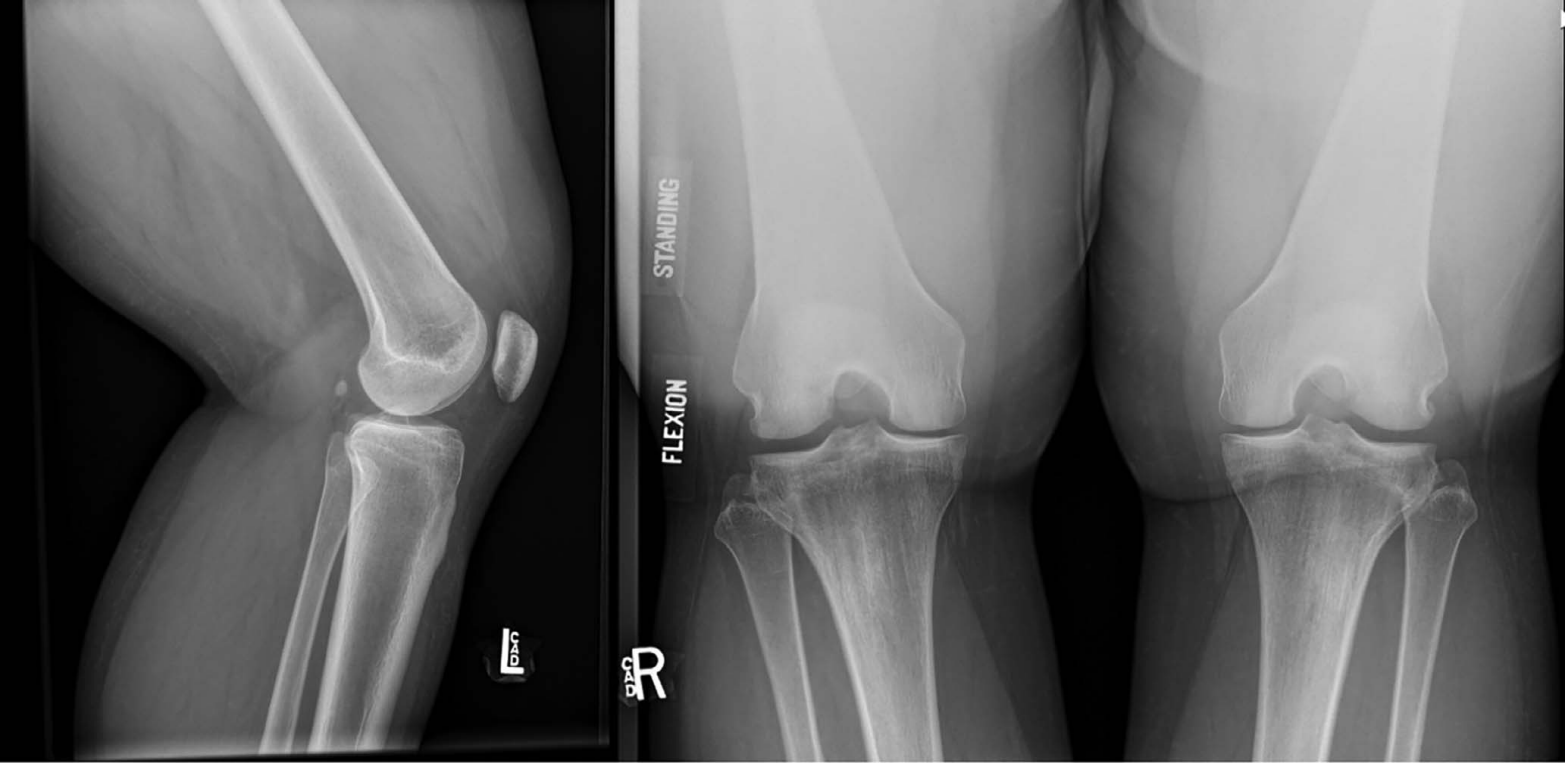
Lateral and standing flexion plain radiographic views of the left knee show no evidence of fracture, dislocation, or degenerative changes.

A 19-year-old male injured his left knee during a collegiate wrestling match and presented to the senior sports orthopedic surgeon 7 days after the injury. The left knee exhibited no erythema, swelling, or deformity, and the patient demonstrated an active range of motion from 0° to 100° of flexion. Physical examination of the left knee revealed a grade 2B Lachman test and an abnormal pivot shift. No fracture, dislocation, or degenerative changes were noted on plain radiographs of the left knee (Figure 1). Magnetic resonance imaging (MRI) completed at an outside facility prior to presentation demonstrated a complete ACL tear with a concomitant full-thickness radial tear at the posterior horn of the lateral meniscus. The patient was unable to provide the treating surgeon with his MRI.

The patient was taken to surgery 9 days after his initial injury. A medial incision of the skin was made with the knee in flexion. The paratenon was incised in the midline to expose the entire width of the patellar tendon. Intraoperatively, the patient's patellar tendon was noted to be 35 mm. The central third of the patellar tendon, with a width of 10.5 mm, was harvested with a 20-mm proximal bone plug and 25-mm distal bone plug. A complex tear of the posterior body of the lateral meniscus was visualized on arthroscopy. Three TRUESPAN meniscal sutures (DePuy Synthes) were applied in vertical and horizontal fashion to stabilize the tear. The medial meniscus was stable to probing with no evidence of occult tears or pathology. Upon visualization of the intercondylar notch, the ACL and posterior cruciate ligament (PCL) were identified. Visualization demonstrated complete tearing of the ACL with an intact PCL. After appropriate preparation, tunnels were drilled in the femur and tibia, and the graft was fixed with interference screws. Following insertion of the BPTB graft, autologous bone graft (saved while preparing bone plugs and tunnels) was impacted into the outer ends of the bone tunnels and into the patellar defect. VICRYL sutures (Ethicon Inc) were placed in inverted fashion to close the parapatellar tendon defect, and a series of VICRYL sutures in figure-eight fashion were used to close the paratenon. A separate incision was created at the lateral epicondylar eminence, and the anterolateral ligament was reconstructed with a semitendinosus allograft. Postoperatively, the patient's knee was placed in a hinged knee brace locked at 10° of hyperextension and allowed flexion to 90°.

**Figure 2.**
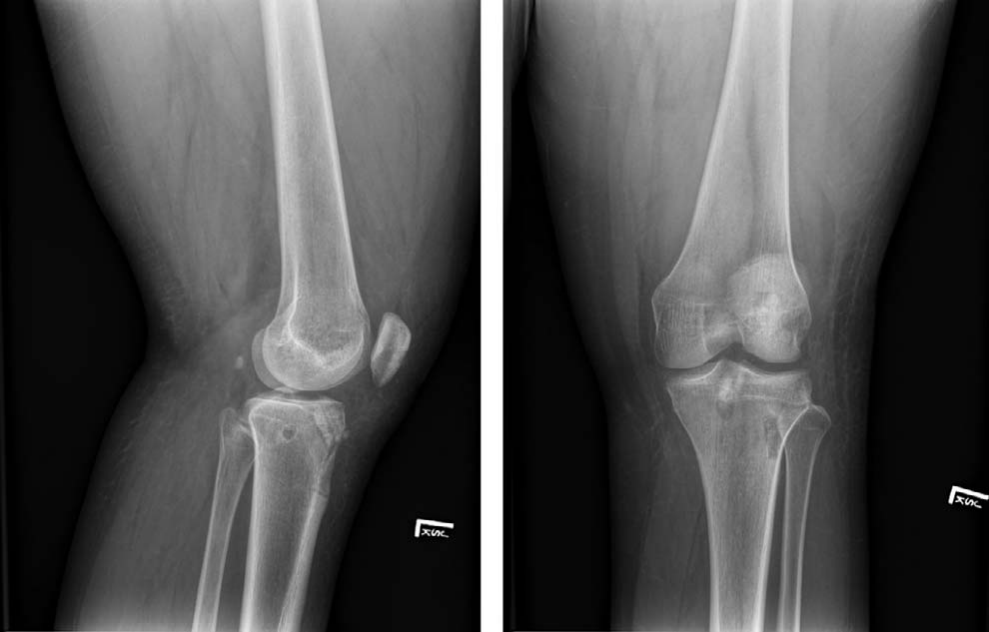
Lateral and standing plain radiographic views of the left knee show well-positioned femoral and tibial tunnels after anterior cruciate ligament reconstruction and normal patellar alignment.

Physical therapy was initiated on postoperative day 3. The patient was allowed toe-touch weight-bearing on crutches with the affected leg locked in 10° of hyperextension. The physical therapy protocol involved quadriceps strengthening, straight leg raises, and active knee extension in a closed-chain fashion. At 2 weeks postoperatively, the patient had progressed to 25% weight-bearing on crutches and planned to continue therapy at his university athletic facility out of state. His radiographs at that time demonstrated well-positioned femoral and tibial tunnels after ACL reconstruction and appropriate patellar alignment (Figure 2).

Approximately 6 weeks after surgery, the patient slipped on ice and fell onto a flexed left knee. He immediately noticed pain, swelling, and ecchymosis; presented to an outside emergency department; and was instructed to remain non–weight-bearing with his brace locked in extension.

**Figure 3.**
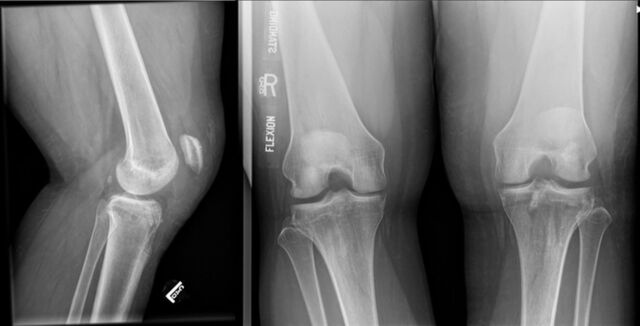
Lateral and standing flexion plain radiographic views of the left knee show interval development of patella alta without additional osseous abnormalities.

**Figure 4.**
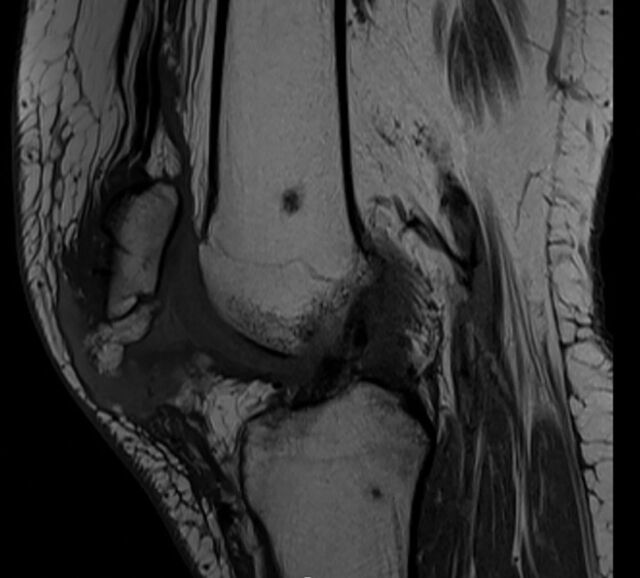
Sagittal T1 magnetic resonance imaging shows a full-thickness tear of the left patellar tendon with retraction.

The patient returned to his primary surgeon's clinic 1 week after the injury. On examination, he was tender to palpation of the patella and the patellar tendon. The extensor mechanism was not intact. Plain radiographs of the left knee demonstrated no acute fracture, patella alta, and soft tissue swelling of the anterior knee (Figure 3). MRI showed a full-thickness tear of the patellar tendon with 4 cm of retraction (Figure 4), a full-thickness tear of the lateral patellar retinaculum, a partial-thickness tear of the medial patellofemoral ligament, and a full-thickness re-tear of the anterolateral ligament at the femoral anchor. The ACL graft was intact.

One week after reinjuring his left knee, the patient was taken back to the operating room for patellar tendon repair. Arthroscopic evaluation of the patellofemoral joint demonstrated severe lateral patellar tilt and severe patellar subluxation with articular cartilage damage. Arthroscopic chondroplasty was performed. The reconstructed ACL and repaired lateral meniscus were both identified and intact. An anterior incision was made, and the patellar tendon was found to have a midsubstance tear with medial involvement. A HEALIX ADVANCE 4.5-mm triple-loaded anchor (DePuy Synthes) was placed in a drill hole made in the inferior pole of the patella. Two ORTHOCORD sutures (DePuy Synthes) were placed in Krackow fashion in the tissue remnant laterally from distal to proximal. A tibialis posterior tendon allograft with a loupe diameter of 9.0 mm was sewn into the anchor in the inferior patella. The free ends of the distal tendon graft were joined with the ORTHOCORD sutures using the SPEEDTRAP Graft Preparation System (DePuy Synthes). Two entry points were made on the tibial tuberosity for the free ends of the tendon graft. Intraoperative fluoroscopy was used to reduce the patella into the anatomic position with the knee in 30° of flexion assessing the inferior pole of the patella and its bisection of the Blumensaat line. With the patella held in position, the sutures from the anchor and the ORTHOCORD sutures were affixed to the tendon graft with a modified Kessler technique. A SwiveLock anchor (Arthrex, Inc) was used to secure the free ends of the graft in the tibial tuberosity. Using a parachute technique, previously placed sutures were passed through the undersurface of an Artelon graft (Artelon). Postoperatively, the patient's knee was placed into a hinged knee immobilizer locked in 10° of extension and allowing flexion to 30°.

The patient began physical therapy on postoperative day 7. He was allowed toe-touch weight-bearing with crutches with the joint immobilized in 10° of extension for the initial 2 weeks. The patient developed a superficial wound 2 weeks following the operation that was drained and dressed with an incisional vacuum-assisted closure. At 4 weeks postoperatively, the patient had achieved 60° of flexion. Imaging performed at 4 weeks postsurgery demonstrated a normal relationship of the patella and femur. The patient's pain level and range of motion continued to improve. At 1-year postoperative follow-up, the patient's knee range of motion was –5° to 130° with 0/10 pain. He exhibited quadriceps weakness (4/5) that was addressed through physical therapy and home exercises. At 2-year postoperative follow-up, the patient reported that his strength had improved, although he still felt it was lacking compared to his preoperative state. He maintained his range of motion and lack of pain and had resumed full activities with wrestling and football.

## DISCUSSION

In 1933, McMaster showed that 50% of a tendon must be compromised before the tendon will rupture under load.^17^ However, more recent studies have demonstrated that several biomechanical and histologic changes occur following removal of the central third of the patellar tendon. LaPrade et al (1997) investigated the patellar tendons of greyhounds.^18^ Six months following removal of the central third of the patellar tendon in the greyhounds, cellularity and collagen fibril size had increased, and fibril packing had decreased. Burks et al (1990) also used a dog model to demonstrate that 3 months following removal of the central third of the patellar tendon, the operated patellar tendon had decreased length, stiffness, and modulus compared to the unoperated patellar tendon in the control group.^19^ Lairungruang et al (2003) found similar results in human cadaveric models.^20^ They investigated the ultimate load-bearing capacity in normal patellar tendons and in patellar tendons after central third removal in cadaveric specimens. The ultimate load of the removal group was almost half that of the normal patellar tendon (2,226.58 N vs 4,365.59 N).^20^ These findings, together with the common slip and fall mechanism of rupture, indicate that following central third removal, the operated patellar tendon lacks the structural integrity of the unoperated tendon.

The most common location of rupture in native patellar tendons is near the inferior pole of the patella^21^ and often occurs after a period of patellar tendinosis.^22^ The surgical management of ruptures in native patellar tendons includes end-to-end repair for midsubstance tears, transosseous tendon repair for proximal avulsions, and suture anchor tendon repairs for distal avulsions.^23^

Both the location and management of harvested patellar tendon ruptures differ from those of native tendons. The most commonly described pattern of rupture of patellar tendons following BPTB graft harvest is the Z-type tear.^3^ Several reports have also described proximal^6,8,11,13^ or distal avulsion.^4,9,11,12,16^ The majority of these tears were repaired with suture anchors or transosseous sutures with or without wire reinforcement. The patellar tendon rupture described in this report was a midsubstance tear. This injury pattern has only been described twice previously,^7,14^ with both reports describing different techniques of surgical repair.

Haskoor and Busconi described a mid-to-distal patellar tendon rupture following a slip and fall accident 6 weeks after BPTB graft harvest.^7^ The authors used a semitendinosus tendon autograft that was fed through the tibial tuberosity inferior to the attachment of the patellar tendon with its distal attachment to the tibia preserved. The autograft was incorporated into the proximal stump of the patellar tendon in a Pulvertaft weave fashion and secured to the tibia with an anchor. Two suture anchors were placed in the patella, and sutures from these anchors were used to connect the proximal and distal stumps. Postoperatively, the patient was placed in a brace locked in extension, and flexion was incrementally increased over 10 weeks.

Milankov Ziva et al reported a midsubstance patellar tendon rupture in a patient jumping while playing a sporting fixture 7 months following BPTB ACL reconstruction.^14^ A BPTB frozen allograft was used to reconstruct the ruptured patellar tendon with bone troughs created in the patella and tibial tuberosity. The remnants of the patellar tendon were also sutured. Multiple wire loops were used to reinforce the reconstruction, running from the upper edge of the patella to a screw in the tibial tuberosity. A continuous passive motion machine was used postoperatively, set to 0° to 45° of flexion and progressing to 90° in the following 2 weeks. After 6 weeks of partial weight-bearing, the patient transitioned to full weight-bearing. The wires and screws were removed 6 months postoperatively because of hardware failure.

We describe a third, distinct technique for repair of a midsubstance rupture following BPTB ACL reconstruction. A tibialis posterior allograft was used to reconstruct the ruptured patellar tendon with a series of anchors fixing the allograft to the patella and tibial tuberosity and sutures incorporating the allograft into the remaining tendon. An Artelon graft was used to reinforce the reconstruction.

The use of an allograft offers several advantages over an autograft. Donor site morbidity is avoided with the use of an allograft, an important consideration for our patient with an already attenuated knee from the prior ACL reconstruction. The use of an allograft also eschews the need for creating multiple osseous defects, as performed by Milankov Ziva et al,^14^ in both the patella and tibia that already have altered anatomy from prior BPTB ACL reconstruction. Another benefit of our technique is that no hardware is implanted. By avoiding metal hardware, the risk of introducing a nidus for infection is reduced. Avoiding hardware also mitigates the need for further surgery for removal of hardware. However, the pros of this technique must be balanced against the early postoperative mobilization that was possible with the wire reinforcement described by Milankov Ziva et al.^14^ In our case, we decided that preventing the need for further surgery outweighed the benefit of early range of motion with a wire loop reinforcement.

The choice of allograft in this report differs from the BPTB allograft previously described.^14^ To our knowledge, no reports document the use of a tibialis posterior allograft for the reconstruction of a patellar tendon rupture following BPTB ACL reconstruction. However, studies have investigated the biomechanical properties of this allograft and the clinical outcomes when used for the reconstruction of other ligaments and tendons. Haut Donahue et al compared the biomechanical properties of tibialis posterior allografts to double-looped semitendinosus and gracilis grafts.^24^ They demonstrated that the structural, material, and viscoelastic properties of a tibialis posterior allograft were comparable or superior to a double-looped semitendinosus and gracilis graft. Tibialis posterior allografts have been shown to be effective in the reconstruction of ruptured tendons and ligaments including ACL,^25^ PCL,^26^ and tibialis posterior tendon.^27^ A polyurethane augmentation device is used to support the allograft during acute healing.

## CONCLUSION

Rupture of the patellar tendon is a rare complication following BPTB ACL reconstruction, and a midsubstance tear is an uncommon presentation of this injury, with only 2 cases previously reported. The use of a tibialis posterior allograft is an effective technique for the management of this injury. This technique avoids donor site morbidity seen in autografts, does not require hardware removal, and confers good functional outcomes.

## Notes

*This article meets the Accreditation Council for Graduate Medical Education and the American Board of Medical Specialties Maintenance of Certification competencies for Patient Care and Medical Knowledge.*
